# Survival and reasons for revision of the uncemented Symax hip stem: A Dutch Arthroplasty Register study

**DOI:** 10.1371/journal.pone.0248483

**Published:** 2021-03-12

**Authors:** Dennis S. M. G. Kruijntjens, Sander M. J. van Kuijk, Liza N. van Steenbergen, Liesbeth M. C. Jutten, J. J. Chris Arts, René H. M. ten Broeke

**Affiliations:** 1 Department of Orthopaedic Surgery, Research School Caphri, Maastricht University Medical Centre, Maastricht, The Netherlands; 2 Department of Clinical Epidemiology and Medical Technology Assessment, Maastricht University Medical Centre, Maastricht, The Netherlands; 3 Dutch Arthroplasty Register (Landelijke Registratie Orthopedische Implantaten), ‘s Hertogenbosch, The Netherlands; Augusta University, UNITED STATES

## Abstract

**Aims:**

Previous studies have already shown early proximal ingrowth, fast osseous integration, and a stable fit of the uncemented Symax hip stem, with excellent clinical and radiographic performance. Aims were to evaluate cumulative revision rates and reasons for revision of the Symax hip stem using Dutch Arthroplasty Register (LROI) data and to assess possible associations between patient characteristics and revision rate of the Symax hip stem.

**Patients and methods:**

All total hip arthroplasties with the uncemented Symax hip stem registered in the LROI between 2007 and 2017 were included (n = 5,013). Kaplan-Meier survival analysis was performed to assess the cumulative 1, 5 and 7-year revision percentages. Cox proportional hazard regression analysis was performed to assess the association between patient and procedural characteristics, and revision arthroplasty of the stem.

**Results:**

Cumulative 1, 5, and 7-year revision rates (with 95% confidence interval (CI)) for revision of any component were 1.5% (CI 1.2%-1.8%), 3.2% (CI 2.7%-3.7%), and 3.8% (CI 3.1%-4.4%) respectively. Cumulative 1, 5, and 7-year stem revision rates of the Symax hip stem were 0.9% (CI 0.6%-1.1%), 1.5% (CI 1.1%-1.9%), and 1.7% (CI 1.3%-2.1%) respectively. Periprosthetic fractures (n = 35) and loosening of the stem (n = 30) were the most common reasons for revision of the stem. Revision of the stem was associated with acute fracture as primary diagnosis (Hazard Ratio (HR) 2.4 (CI 1.3–4.3)), or history of a previous surgery to the affected hip (HR 2.7 (CI 1.4–5.2)).

**Conclusion:**

This population-based registry study shows revision rates for the Symax hip stem comparable to those for best performing uncemented total hip arthroplasties in the Netherlands. Primary diagnosis of an acute fracture, and history of previous surgery on the affected hip, were significantly associated risk factors for revision of the Symax hip stem, and we discourage the use of the Symax hip stem in these patients.

## Introduction

The uncemented Symax hip stem was developed as an optimization of the uncemented Omnifit hip stem [1]. The design considers the geometry of the stem, surface texture, and type and extent of the osseointegrative coating [2, 3]. Previous studies have proven early ingrowth with histological and histomorphometric analyses on retrieved implanted Symax hip stems, exclusively into the proximal part of the stem, as a result of the BONIT-hydroxyapatite (HA) coating [2]. Furthermore, a 2-year follow-up dual-energy X-ray absorptiometry (DEXA) study showed improved bone remodelling compared to the Omnifit hip stem [4]. The stem showed early stabilization in 2 independent RSA studies, and excellent clinical outcomes in a 5-year clinical and radiographic follow-up study [5–7]. In a Danish implant registry study that included 1,055 total hip arthroplasties (THAs) in a single centre, the estimated 6.5-year survival rate of the Symax hip stem with all-cause revision as the endpoint was 97.5% (CI 96.6%-98.3%) [8]. Most common reasons for revision surgery in that study were periprosthetic fractures (n = 11) and recurrent dislocations (n = 10) [8].

The current study illustrates the ‘phased introduction’ of the uncemented Symax hip stem [9, 10]. The idea of a stepwise clinical introduction of a new orthopaedic implant is to ensure quality of orthopaedic implants, and thus patient safety [10]. The Dutch Arthroplasty Registry (LROI) is a nationwide population-based registry covering all hospitals in the Netherlands, which was initiated by the Dutch Orthopaedic Association in 2007 [11]. The LROI contains prospectively collected data on primary and revision arthroplasty. Patient characteristics are recorded at the time of the primary procedure. In 2017, the registry completed its first 10 years of data collection, prompting us to evaluate the cumulative revision rates and the reasons for revision of the uncemented Symax hip stem in this first decade of registering.

Primary aim of this study was to evaluate the cumulative revision rates and the reasons for revision of the uncemented Symax hip stem in total hip arthroplasties 2007–2017 in the Netherlands. Secondary aim was to determine the associations between patient characteristics and reasons for revision. We hypothesized that the Symax hip stem meets the benchmark criteria for best prostheses following the National Institute for Health and Care Excellence (NICE) and Orthopaedic Data Evaluation Panel (ODEP) guidelines. The NICE guidance states that the best protheses should demonstrate a ‘benchmark’ revision rate of 5% or less at 10 years, or, as a minimum, a 3-year revision rate consistent with this benchmark [12]. The ODEP-ratings consists of a number and a letter, and a star (optional) [13]. The number represents the number of years for which the product’s performance has been evidenced. The letter represents the strength of the evidence (data). Letter ‘A’ represents strong evidence, and letter ‘B’ represents acceptable evidence. A star will be added when a benchmark replacement rate is defined of less than 1 in 20 (5%) at 10 years.

## Patients and methods

### Registry

The Dutch nationwide LROI database contains 99% of all primary total hip arthroplasties and 98% of revision hip arthroplasties [14]. It contains information on patient characteristics such as age, gender, and general health (ASA score). Since 2014, body mass index (BMI), smoking behaviour, orthopaedic vitality (i.e. Charnley score), and postal code were added to the database. Furthermore, hospital of surgery, anonymized (encrypted) surgeon, type and date of surgery, indication for surgery, surgical approach, fixation, and prosthesis characteristics (as specified below) were also registered. Implant information was retrieved from the LROI implant library, is based on the article number and includes among others name and type of the prosthesis, material, and femoral head size [11]. Finally, data from the LROI were matched with the encrypted citizen service number of the national insurance database on healthcare (Vektis 2017) in order to obtain information on the vital status (or date of death) of registered patients [11, 14].

### Data collection

All THAs in the Netherlands with an uncemented Symax hip stem registered in the LROI in the period between 2007 and 2017 were included. It is possible for a patient to be registered twice, if both hips were operated (bilateral THA). A primary THA was defined as the first time a total hip prosthesis is implanted to replace a hip joint. Revision arthroplasty was defined as any change (replacement, removal, or addition) of one or several components of the joint prosthesis [14]. The encrypted personal citizen number allowed linkage of revision arthroplasty procedure to the primary procedure. Reasons for revision surgery were categorized as infection, cup/liner wear, periprosthetic fracture, dislocation, loosening femoral component, loosening acetabular component, periarticular ossifications, and other. It was possible to register more than one reason for revision.

### Prosthesis

**Fig 1:**
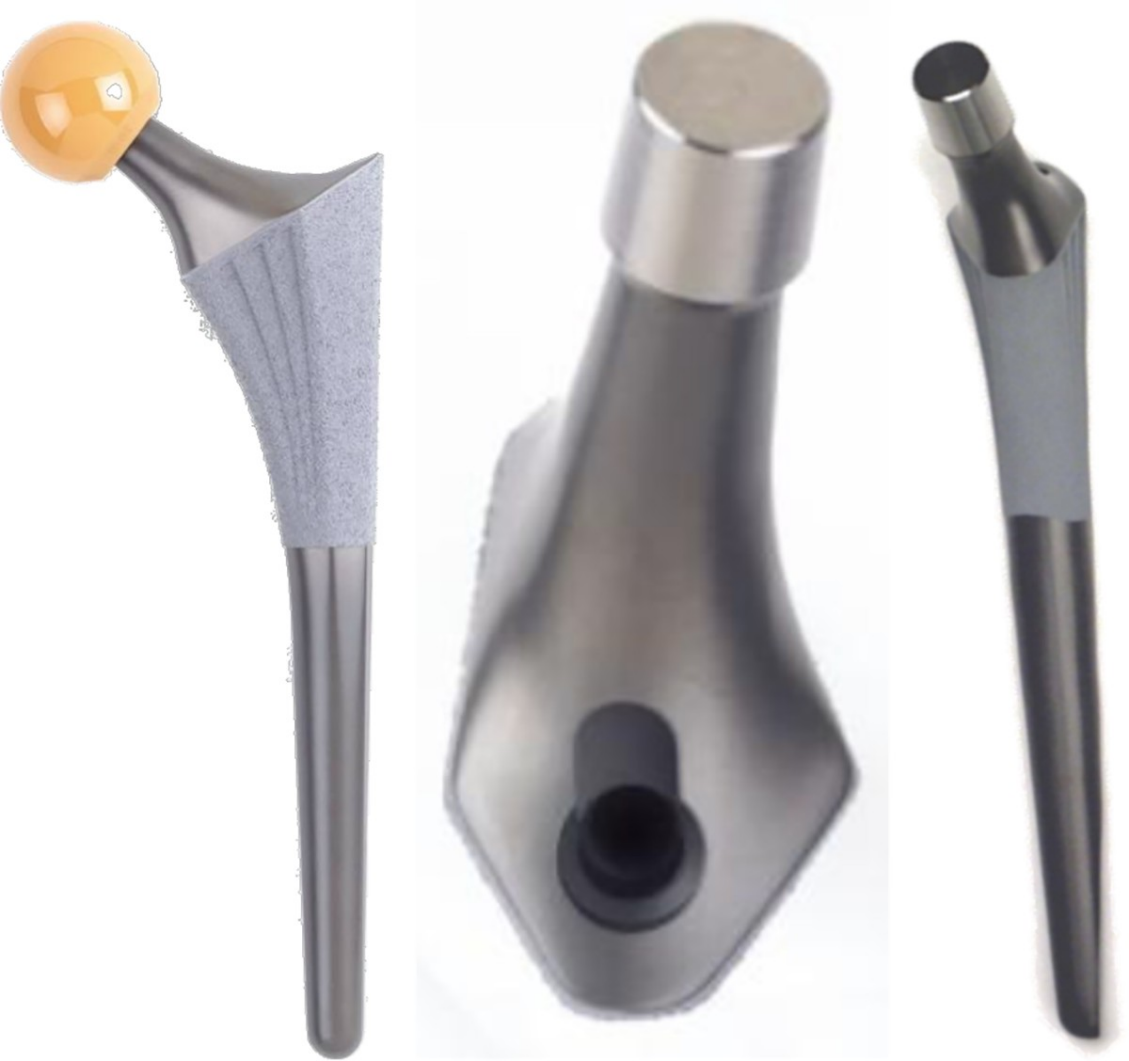
Design features of Symax hip stem. Illustrating the anatomically anteverted proximal geometry, with the BONIT-HA coating; and the straight distal part with the DOTIZE surface treatment and a posterior chamfer.

The Symax hip stem is an uncemented design forged from Ti6Al4V alloy (CE 545074). Primary mechanical stability is provided by anatomical metaphyseal geometry (Fig 1). The hip stem features a size-dependent anteversion, neck length and offset, with a CCD angle of 128°. Secondary biological stability is accomplished by fast osseous integration due to the BONIT-HA coating on the metaphyseal part of the stem. The BONIT-HA is an electrochemically deposited, biomimetic hydroxyapatite (HA) coating on top of a commercially pure titanium plasma spray (TPS) layer. It is deposited by low-temperature precipitation, is thin (10–20μm), and has a 3D surface with high porosity (60%) and pore interconnectivity [2, 4]. The coating is fully resorbable and is substituted by bone for about 99% [15]. The anodization surface treatment, DOTIZE, applied on the distal part of the stem, is an electrolytical conversion of the native oxide film on titanium surfaces into a thicker and denser titanium oxide. It shows anti-galling properties, reduces protein adsorption with 19% and bone apposition compared to an untreated titanium alloy [2, 4].

### Statistical analysis

Descriptive statistics were used for the presentation of baseline characteristics of the cohort at the time of primary procedure. Results were reported as absolute value and percentage. Follow-up started on the date of the primary surgery, and ended at revision, death, or end of the study period (1^st^ January 2017), whichever came first. Kaplan-Meier survival tables with 95% confidence intervals (CI) were used to estimate the cumulative 1, 5, and 7-year revision percentages. In order to assign the revision rates according to the NICE and ODEP, and to be able to compare the revision rates to other prostheses, the cumulative revision percentages for both revision of any component (e.g. cup, insert, stem, and/or head), and for revision of the uncemented Symax stem in particular, were estimated. Cox proportional hazards regression analysis was performed to assess the association between patient and procedure characteristics, and the need for revision arthroplasty. We used univariable and multivariable Cox regression analysis to examine the association between potential predictors and the outcome. All potential predictors were entered into the multivariable model. Using backward elimination on the significance of hazard ratios, we deleted non-significant variables from the model. As the number of events was lower than suggested by generic rules of thumb for multivariable modelling (i.e.: at least 10 events-per-variable), the analyses were considered exploratory. Since the amount of clustering in the data due to bilaterality was considered to be very low (<10%) we chose not to use models designed for clustered data such as the frailty model [16]. P-values of 0.05 and lower will be considered to indicate statistical significance.

### Potential conflicts of interests

No conflict of interest was declared and no personal funding was received. No research grant was received.

## Results

Between January 1^st^ 2007 and January 1^st^ 2017, a total of 5,013 THAs were implanted in 4,593 patients (420 bilateral) in the Netherlands. The mean age at surgery was 67.4 years (range 14–97 years), 62% were female patients, and in 83% of the patients the primary diagnosis was osteoarthritis (Table 1). The median follow-up was 5.2 years (range 0–10 years).

**Table 1:** Patient and procedure-related baseline characteristics of the study cohort.

Total		n = 5013	
			% of subgroup
**Gender**			
	Female	3072	62%
	Male	1918	38%
	Missing	23	
**Age groups (years)**		
	< 49	319	6%
	50–59	687	14%
	60–69	1735	35%
	70–79	1647	33%
	> 80	619	12%
	Missing	6	
**ASA classification**		
	ASA I	1123	23%
	ASA II	2903	60%
	ASA III-IV	844	17%
	Missing	143	
**Diagnosis**			
	Osteoarthritis	4143	83%
	Acute fracture	443	9%
	Osteonecrosis	157	3%
	Other	270	5%
**Fixation**			
	Uncemented	4756	95%
	Reversed hybrid	257	5%
**Previous surgery on affected hip**		
	Yes	280	6%
	No	4523	91%
	Unknown	144	3%
	Missing	66	
**Approach**			
	Posterolateral	1985	40%
	Direct lateral	2159	43%
	Anterolateral	804	16%
	Other	65	1%

### Revision rates

**Fig 2:**
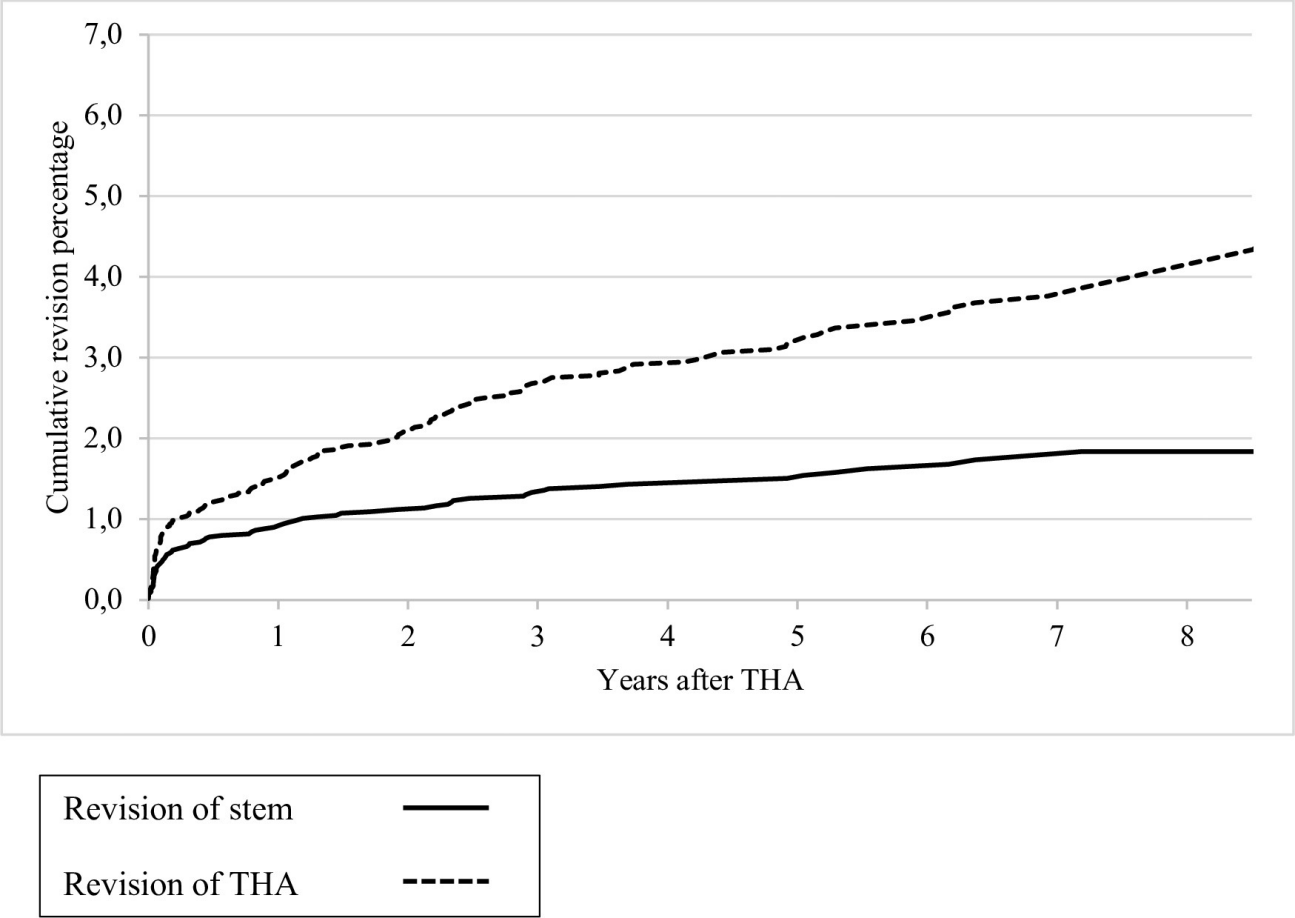
Kaplan-Meier cumulative revision percentages for revision of stem and THA.

The cumulative 1, 5, and 7-year overall revision rates (with 95% CI) for revision of any component were 1.5% (1.2%-1.8%), 3.2% (2.7%-3.7%), and 3.8% (3.1%-4.4%) respectively (Fig 2). The cumulative 1, 5 and 7-year stem revision rates (with 95% CI) of the Symax hip stem were 0.9% (0.6%-1.1%), 1.5% (1.1%-1.9%), and 1.7% (1.3%-2.1%) respectively (Fig 2).

### Reasons for revision of the stem

In total, 76 patients underwent a revision of the stem. Of this subgroup, the mean age at surgery was 67.4 years (range 36–90 years), 66% were female patients, and in 76% of the patients the primary diagnosis was osteoarthritis (Table 2). Periprosthetic fractures (n = 35) and loosening of femoral component (n = 30) were the most common reasons for revision (Table 3). Since multiple reasons for revision were allowed, 12 patients were registered for both periprosthetic fractures as for loosening of the femoral component.

**Table 2:** Patient and procedure-related baseline characteristics of the revision cohort.

Total		n = 76	
			% of subgroup
**Gender**			
	Female	50	67%
	Male	25	33%
	Missing	1	
**Age groups (years)**		
	< 49	9	12%
	50–59	9	12%
	60–69	23	30%
	70–79	22	29%
	> 80	13	17%
	Missing	6	
**ASA classification**		
	ASA I	11	15%
	ASA II	48	66%
	ASA III-IV	14	19%
	Missing	3	
**Diagnosis**			
	Osteoarthritis	58	76%
	Acute fracture	13	17%
	Osteonecrosis	2	3%
	Other	3	4%
**Fixation**			
	Uncemented	72	95%
	Reversed hybrid	4	5%
**Previous surgery on affected hip**		
	Yes	10	13%
	No	63	84%
	Unknown	2	3%
	Missing	1	
**Approach**			
	Posterolateral	33	43%
	Direct lateral	29	38%
	Anterolateral	13	17%
	Other	1	1%

**Table 3:** Reasons for revision of the Symax hip stem (n = 76).

	**n**	**% of revisions**	**% of THAs**
**Infection**	9	12%	0.2%
**Cup / liner wear**	1	1%	0.0%
**Periprosthetic fracture**	35	46%	0.7%
**Dislocation**	7	9%	0.1%
**Loosening femoral component**	30	40%	0.6%
**Loosening acetabular component**	2	3%	0.0%
**Periarticular ossification**	2	3%	0.0%
**Other**	15	20%	0.3%

Values represent the numbers of revision of stems, percentages (%) of the total number of revisions of stems (n = 76), and percentages (%) of the total number of THAs. One patient may have more than one reason for revision. As such, the total proportion is over 100%. Note: there are 25 revision procedures with more than one reason for revision.

### Risk factors for revision of the stem

**Fig 3:**
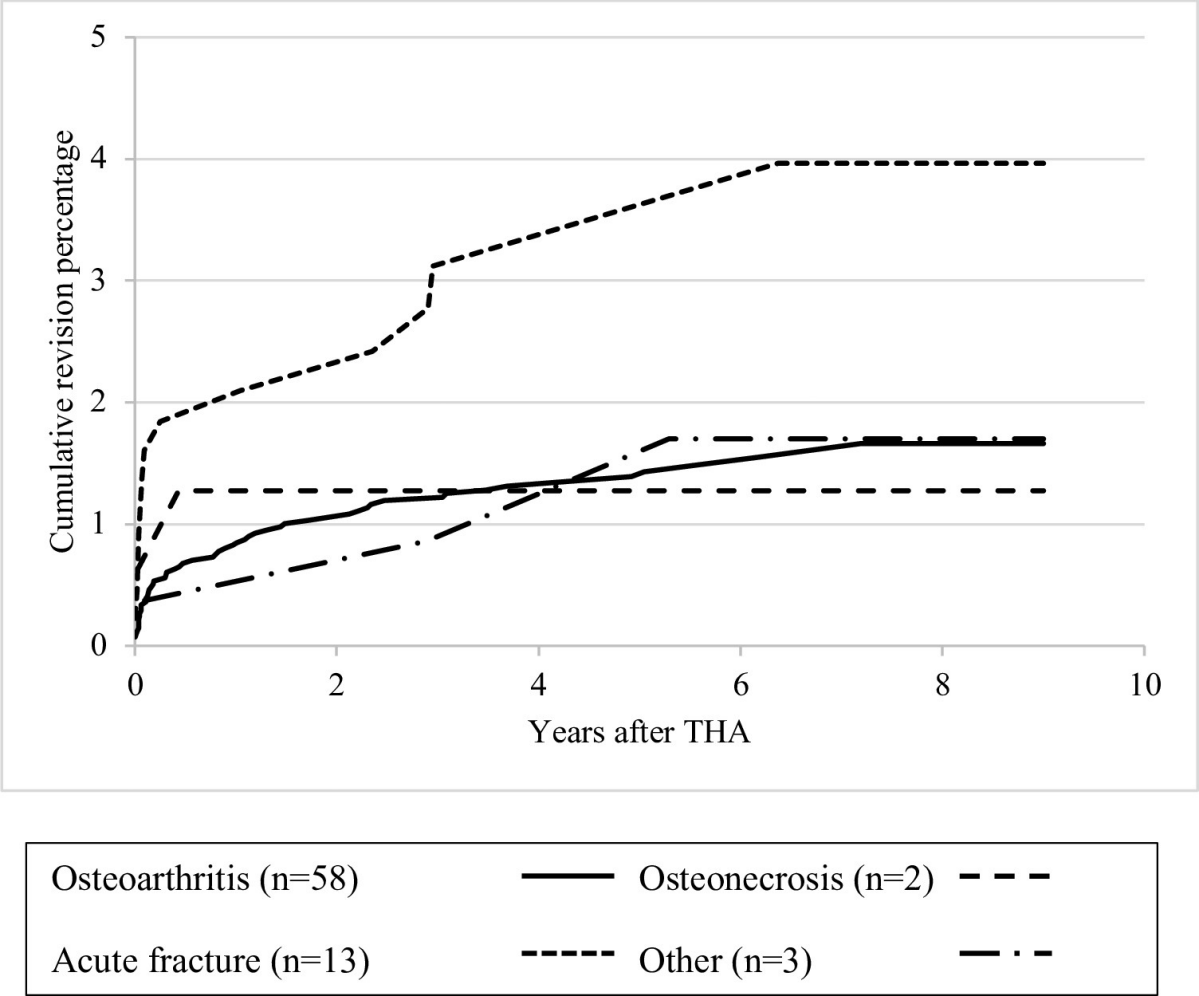
Kaplan-Meier cumulative revision percentages for revision of the stem according to primary diagnosis.

**Fig 4:**
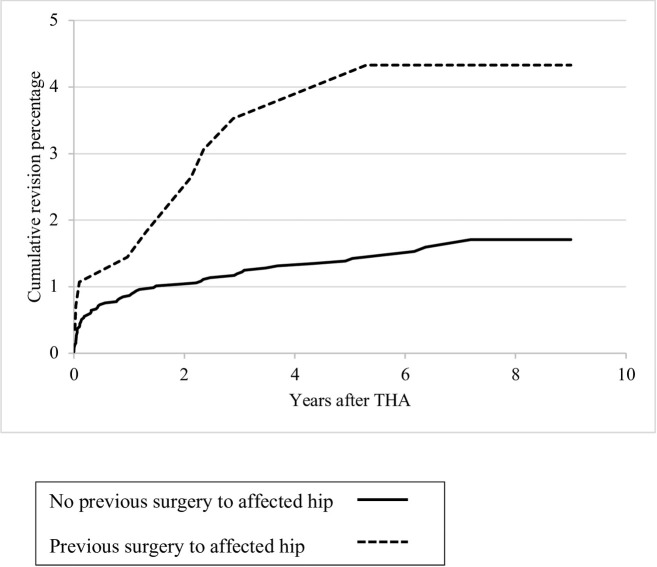
Kaplan-Meier cumulative revision percentages for revision of the stem according to previous surgery to the affected hip.

An acute fracture as primary diagnosis or a previous operation of the affected hip were risk factors for revision of the stem (Table 4) in both the unadjusted and multivariable model. The proportional hazard assumptions were not violated for the evaluated risk factors. Stratified analyses for revision of the stem according to primary diagnosis and previous surgery on the affected hip showed a 5-year revision rate of the stem of 3.1% (1.4%-4.9%) for THAs for acute fractures (n = 443), compared to 1.4% (1.0%-1.8%) for THAs for osteoarthritis (Fig 3). THAs in patients with a previous surgery (n = 280) on the affected hip showed a revision rate of the stem of 3.5% (1.3%-5.4%) compared to 1.4% (1.0%-1.7%) for patients without a previous surgery in the affected hip (Fig 4).

**Table 4:** Cox proportional Hazard Ratios (HR, with 95% Confidence Intervals (CI)) to assess the association between patient and procedure characteristics for revision of the Symax hip stem.

	**HR (95% CI)**
Gender	
* Male	Reference
* Female	1.2 (0.8–2.0)
Age (in years)	1.0 (1.0–1.0)
ASA classification	
* I-II	Reference
* III-IV	1.2 (0.7–2.2)
Diagnosis	
* Osteoarthritis	Reference
* Acute fracture	2.4 (1.3–4.3)
* Osteonecrosis	1.0 (0.2–4.1)
* Other	0.8 (0.3–2.7)
Fixation	
* Uncemented	reference
* Reversed hybrid	1.0 (0.4–2.8)
Previous surgery	
* No	reference
* Yes	2.7 (1.4–5.2)
Approach	
* Posterolateral	reference
* Direct lateral	0.7 (0.4–1.2)
* Anterolateral	0.9 (0.5–1.6)
* Other	0.8 (0.1–6.1)

## Discussion

In this population-based National Dutch Implant Registry study, we evaluated the cumulative revision rates and the reasons for revision of the Symax hip stem. The cumulative 1, 5, and 7-year revision rates of the Symax THA were comparable to the cumulative revision rates for uncemented THAs in the Netherlands in 2007–2016, as these were 1.6% (1.5%-1.6%), 3.4% (3.3%-3.6%), and 4.3% (4.1%-4.4%) respectively [14]. Our study population was comparable to the total group of patients with uncemented THAs in the Netherlands for age, gender distribution, and diagnosis. The cumulative revision rates are also in line with the median 6.5-year survival rates of the Symax hip stem with all-cause revision as the endpoint of 97.5% (96.6%-98.3%) in Vejle, Denmark [8]. The NICE recommends only those hip prostheses which have (projected) revision rates of 5% or less at 10-year clinical follow-up [12]. The ODEP was initiated in the UK in 2002 and independently evaluates hip prostheses according to the NICE guidelines [13]. The ODEP assigns each prosthesis design a benchmark rating, so that they can be compared to other prostheses that meet the NICE guidelines. In conclusion, the Symax hip stem meets the benchmark criteria of the NICE guidance, and can be classified as a 7A according to the ODEP criteria [12, 13].

The most common reasons for revision of the Symax hip stem were loosening of femoral component and periprosthetic fractures. (Aseptic) loosening of the Symax hip stem occurred in 0.4% of the study population. On the contrary, no revisions caused by aseptic loosening of the Symax hip stem were reported in a Danish single-centre study [8]. Furthermore, early stabilization of the Symax hip stem was observed already after 4 weeks in a 2-year RSA study, and minimal subsidence of the Symax stems was measured in a 3-year EBRA-FCA study [7, 17]. Another RSA study of the Symax hip stem also showed predominantly Y-translation and Y-rotation at 3 months, and zoledronic acid had no significant effect in this femoral stem migration [6]. In this randomized controlled trial zoledronic acid maintained periprosthetic bone mineral density (BMD) during the first 12 months, while in the control group the expected loss of BMD occurred. Thereafter, periprosthetic BMD decreased even in the zoledronic acid group in Gruen zone 7, but remained 14.6% higher than in the placebo group at 4-years follow-up [6]. A study comparing BMDs around the uncemented Symax and Omnifit hip stems showed values that were statistically significant in favour of the Symax hip stems [4]. All these studies indicate a minimal risk of aseptic loosening for the Symax hip stem.

The prevalence of revision due to periprosthetic fracture for the Symax stem at 10.2-years was 1.0%, which is slightly more than in our current study (0.7%) [8]. Thien et al. found in their 2-year Nordic Arthroplasty Register Association database that the incidence of revision due to periprosthetic fracture was 0.47% for cementless THAs (Bi-Metric, CLS Spotorno, Corail, ABG I and II) [18]. Nearly all fractures occurred during the first 6 months. The exact reason for the relatively high prevalence of periprosthetic fractures of the Symax hip stem compared to other uncemented stems is unclear. 20% of the periprosthetic fractures of the stem were in patients who had a primary diagnosis of an acute fracture, and 6% because of late post-traumatic osteoarthritis. 60% of the periprosthetic fractures of the stem occurred during the first 2 months, which is in line with the findings of Thien et al. [18]. These early fractures could be initiated during the primary surgery as minimal fissures and progress to significant fractures during rehabilitation [18]. This again confirms that results of uncemented stems, implanted for proximal femoral fractures are worse compared to those implanted for osteoarthritis and avascular necrosis. Bergschmidt et al. discontinued using the Symax hip stem because of subsidence of more than 10mm in 2 patients and 3 intraoperative periprosthetic fractures outside their study population [19]. In a clinical DEXA study comparing the Symax to the Omnifit hip stem, improved stress transfer from the bone to the implant in the important posterior and medial areas was proven for the Symax hip stem. This led to improved preservation of periprosthetic bone compared to other proximally, and entirely porous or HA-coated stems, which might lead to a decrease in periprosthetic fractures and aseptic loosening [3].

The secondary aim of this current study was to estimate the associations between patient characteristics and reasons for revision. As mentioned above, a primary diagnosis of acute fracture was associated with a statistically significant increased risk for revision of the stem. This is in line with the results of a Danish implant register study in which uncemented femoral components were associated with a statistically significant increased risk of early periprosthetic femoral fractures (relative risk (RR) 4.1, CI 2.3–7.2), especially in elderly (RR 1.4 per 10 years, CI 1.2–1.6), female (RR 0.6, CI 1.1–2.2) and osteoporotic patients (RR 2.8, CI 1.6–4.8) [20]. Furthermore, Thien et al. concluded in their study on 439,629 THAs in the Nordic registry that cementless stems should be avoided when advanced age, female gender and a femoral neck fracture are present [18]. Besides this, several other studies have proven that there are more complications with uncemented than cemented femoral stems in both THA and hemiarthroplasty for displaced femoral neck fractures [21, 22]. However, there are several uncemented stem designs. Carli et al. identified a substantial variability in the performance of uncemented stem designs, with an increased risk for periprosthetic fractures in both type 1 (‘Single-wedge’ or ‘blade-type’) stems and type 2 (‘double-wedge’ or ‘fit-and-fill’) stems [23]. Statistically significant, and clinically relevant, lower rates of periprosthetic fractures were shown in type 6 (anatomical) stems, and a group consisting of type 3 (tapered round, spline, rectangle) and type 4 (cylindrical, fully coated) stems [23]. As the uncemented Symax hip stem can be classified as a type 2 stem, it is advised not be used for acute femoral fractures.

A previous operation to the affected hip was also a statistically significant, and clinically relevant, risk factor for revision of the femoral stem. This is in line with the results of a systematic review and meta-analysis in which THA after failed osteosynthesis (salvage or conversion THA) was associated with more complications compared to primary THA for intracapsular femoral neck fractures [24]. Although the optimal treatment for intracapsular femoral neck fractures remains debatable in independently mobile patients, fixation failure occurs in about 30% of these patients [25]. So, the outcome of conversion THA must be considered thoroughly. Other studies about conversion THA after prior proximal femoral trauma have also shown heterogeneous results depending on the initial fracture and fixation type. Conversion of prior intertrochanteric fracture fixation has been associated with poorer outcomes compared to prior femoral neck fractures [26]. Conversion from prior intramedullary fixation is more complex and challenging than from prior sliding hip screw, due to the increased damage to the medullary canal and the hip abductor mechanism [27]. Therefore, one has to take into account that for the Symax hip stem, as well as for other uncemented designs, the use is associated with a higher complication risk in case of previous surgery to the hip.

The strength of this study is that it is the first nationwide study about the Symax hip stem using data from the LROI Dutch Arthroplasty Register. The data is prospectively collected, the sample size is large, and the follow-up is complete. As it is a nationwide register study the data is generalizable. A limitation of this study is that not all implanted Symax hip prostheses are incorporated in the registry, because the registry started in 2007, while the Symax hip stem was introduced in 2004. The number of registered patient characteristics was limited in this registry. For example, alcohol and drug use, fluid and electrolyte disorders, and peripheral vascular disorders are not included, while these are known to influence revision rates [28]. Smoking behaviour, BMI, Charnley score, and postal code were added since 2014 to the LROI, so these were limited available for the current population. Furthermore, not all of the complications that have occurred are registered, since only revision procedures were included in the LROI. This probably underestimates the total number of complications. Nevertheless, this is the largest cohort of patients available in the Netherlands, providing the best nationwide evidence on the survival and revision rate of the Symax hip stem.

## Conclusions

In summary, overall cumulative revision rates of the Symax hip stem are comparable to the overall cumulative revision rates for best performing uncemented THAs in the Netherlands at every follow-up up to 9 years follow-up. The Symax hip stem meets the benchmark criteria of the NICE guidance, and it can be classified as a 7A according to the ODEP criteria. Periprosthetic fractures and loosening of the femoral component were the most commonly registered reasons for revision of the femoral component. An acute fracture as the primary diagnosis and a history of previous surgery on the affected hip were statistically significant, and clinically relevant, associated risk factors for revision of the Symax hip stem, and we discourage the use of the Symax hip stem in these patients.
